# Obituary

**Published:** 1904-07-15

**Authors:** 


### OBITUARY.

### ACTION REGARDING THE DEATH OF DR. TAFT.

The following memorial resolution was adopted by the Cincinnati Odontological Society at its November meeting:

The members of the Cincinnati Odontological Society wish to record this expression of their regret and sorrow at the death of Dr. J. Taft, a charter member of this organization. Up to the time of his removal to Michigan, he was regular in his attendance upon the monthly meetings of the society, and always took an active part in the proceedings.

His frequent communications to the society, as well as his discussion of the subjects brought before the body, were notable, scientific and practical. contributions. Dr. Taft's long and varied experience as a foremost practitioner of dentistry in this city and his ability as a writer and speaker, made him a most desirable member of any association of dentists, and the members of the Odontological Society always felt they were helped and enlightened when he was present at their meetings.

Dr. Taft was also an example to all our members, because of his high professional standards which he set before them and which he himself always strived to practice.

The members of the Odontological Society wish to convey to Mrs. Taft, his daughter, Mrs. A. T. Edwards, and his sons, Drs. William and Alfonso Taft, expressions of sympathy in their sorrow and bereavement.

Signed, H. A. Smith,
M. H. Fletcher,
J. R. Callahan,
*Committee.*

### MEMORIAL TO DR. TAFT.

WHEREAS, In the death of Dr. Jonathan Taft, whose death occurred at Ann Arbor, Mich., October 15th, 1903, the dental profession has lost a most valuable member, one who gave generously of his knowledge for the advancement of his ideals; a noble manhood, ideal operations and faithful services; a man unselfish, gifted, kind and true, using his God-given talents for a noble purpose—serving man, and one whose fidelity and Christian character must ever be an example to the coming generations of an ideal dentist.

*Be it Resolved:* That we his followers and members of the Northern Ohio Dental Association, in annual convention assembled at Cleveland, June 7th to 9th, 1904, express and record our appreciation of his noble life and generous contributions to the dental profession, and urge upon every member to live the life this gifted man lived, in order to bring to pass his conception of an ideal dental profession.

Signed, C. R. BUTLER,

CORYDON PALMER,

F. S. WHITSLER,

*Committee.*

C. D. PECK, *Recording Secretary.*

